# VGF: a biomarker and potential target for the treatment of neuropathic pain?

**DOI:** 10.1097/PR9.0000000000000786

**Published:** 2019-09-19

**Authors:** Nadia Soliman, Kenji Okuse, Andrew S.C. Rice

**Affiliations:** a Pain Research, Department of Surgery and Cancer, Imperial College London, London, United Kingdom; b Department of Life Sciences, Imperial College London, London, United Kingdom

**Keywords:** VGF, Neuropathic pain, Neuropeptide, Sensory neurons, Macrophages, Microglia, TLQP-21, C3aR1, gC1qR

## Abstract

Neuropathic pain (NP) remains an area of considerable unmet medical need. A persistent challenge in the management of NP is to target the specific mechanisms leading to a change from normal to abnormal sensory perception while ensuring that the defensive pain perception remains intact. Targeting VGF-derived neuropeptides may offer this opportunity. VGF was first identified in 1985 and is highly expressed after nerve injury and inflammation in neurons of both the peripheral and central nervous system. Subsequent studies implicate the *vgf* gene and its products in pain pathways. This narrative review was supported by a systematic search to identify, select, and critically appraise all relevant research investigating the role of VGF-derived neuropeptides in pain pathways. It predominantly focuses on in vivo investigations of the role of VGF in the initiation and maintenance of NP. VGF expression levels are very low under normal physiological conditions and nerve injury results in rapid and robust upregulation, increasing mechanical and thermal hypersensitivity. The identification of the 2 complement receptors with which VGF neuropeptides interact suggests a novel interplay of neuronal and immune signalling mediators. The understanding of the molecular mechanisms and signalling events by which VGF-derived active neuropeptides exert their physiological actions is in its infancy. Future work should aim to improve understanding of the downstream consequences of VGF neuropeptides thereby providing novel insights into pain mechanisms potentially leading to the identification of novel therapeutic targets.

## Abstract 2

Supplemental Digital Content is Available in the Text.

## 1. Background

**Figure 1.**
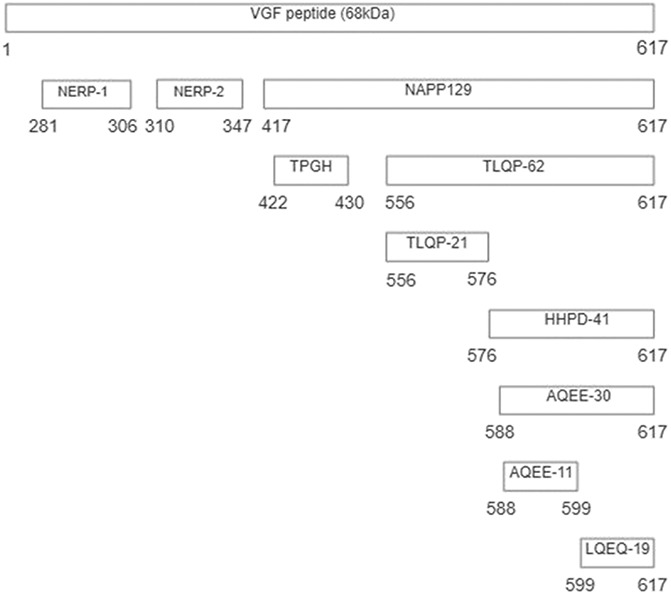
The *vgf* gene and its derived peptides. The VGF polypeptide is the precursor of several biologically active peptides. The gene contains specific amino acid sequences, as shown, of the pro VGF polypeptide and C-terminal VGF-derived peptides, which are highly conserved between species.

VGF (nonacronymic) is a granin-like neuropeptide precursor whose expression is robustly regulated by neuronal lesions and growth factors.^9,34,55^ VGF-derived peptides have a functional role in several disorders including obesity, dementia, depression, and pain. This review focuses on the role of VGF in pain pathways. VGF was first identified due to its rapid induction in PC12 cells after treatment with nerve growth factor (NGF).^22,54,86^ Subsequent studies have identified numerous neurotrophins that also upregulate VGF expression including brain-derived neurotrophic factor (BDNF) and neurotrophin-3, in targets such as cortical or hippocampal neurons.^1^ The *vgf* gene encodes a precursor protein of 615 (human) and 617 (rat and mice) amino acids.^55,87^ The VGF precursor protein sequence is highly conserved among rats and mice, with only 21 out of 617 amino-acid substitutions, none of which occur at the C-terminus. The precursor protein contains approximately a dozen cleavage sites and cleaves different peptides with specific neuronal bioactivities. Functional effects have been reported for several proteolytic products contained within the C-terminal 62 amino acid portion of VGF. Several of these VGF-derived peptides have been identified and are named by the first 4 N-terminal amino acids and their total length (eg, TLQP-62, TLQP-21, HHPD-41, AQEE-11, and LQEQ-19, reviewed in Ref. 55) (Fig. 1). VGF has a tissue-specific pattern of expression and is robustly regulated and synthesised in immune cells, neuronal cells, and neuroendocrine cells of both the central and peripheral nervous system.^14,15,18,54,79,86,94^ In the peripheral nervous system, VGF is highly expressed in both neurons of the sympathetic ganglia and dorsal root ganglia (DRG) of primary sensory neurons.^33^ In the central nervous system (CNS), robust expression of VGF mRNA has been detected in the adult rat spinal cord and brain.^35,93,94^

After peripheral tissue damage, nerve injury, or inflammation, changes to protein expression and functional properties of sensory neurons leads to altered nociceptive processing in the spinal cord.^17,108,110^ Neuropathic pain (NP) is defined as “pain caused by a lesion or disease of the somatosensory system”^49,99^ and can result from various causes including disease, drugs, spinal cord injury, and nerve trauma. The symptoms of NP are characterised by pain produced in the absence of stimulation of nociceptor and spontaneous pain. Neuropathic pain can occur in the context of sensory loss (anaesthesia dolorosa) or sensory gain hyperalgesia, hyperpathia, and allodynia, which are triggered and maintained by a combination of peripheral and central mechanisms.^7^ Current animal models of injury-related or pathological persistent pain only reflect the sensory gain component. The occurrence of sensory abnormalities (gain and loss) varies between and within pathophysiological conditions.^8,59,66^ Each condition can be associated with multiple and various underlying pain generating mechanisms, which are not fully understood. Current therapies have limited efficacies and unwanted side effects and therefore, there remains a large unmet medical need to improve understanding and identify novel therapeutic targets.^36,38^

*In vivo* evidence implicates the *vgf* gene and its products in pain signalling pathways. Increased levels of VGF mRNA and protein have been measured in the DRG and spinal cord in animal models of pathological persistent pain.^24,71,82,100^ The DRG contain the cell bodies of nociceptors and are the neurons that convey nociceptive signals. Therefore, they have been the focus of many gene expression studies in pain models because transcriptional changes affect neuronal sensitivity. The VGF polypeptide is sorted into secretory granules and stored in the sensory terminals, processed into small peptides by endoproteolytic cleavage, and released on depolarisation activation.^55,82^ Administration of exogenous VGF-derived peptides is reported to result in both mechanical and thermal hypersensitivity.^21,71,82^ Similarly, several authors report that endogenous VGF-derived peptides contribute to both inflammatory and nerve-injury–induced hypersensitivity.^21,32^ Despite the regulation of VGF mRNA by peripheral nerve injury, the role of VGF in the initiation, development, and maintenance of NP remains to be identified.

The most studied VGF-derived peptide is TLQP-21. It has been identified as a ligand for 2 receptors: the complement 3a receptor (C3aR1) and the globular head of the complement component C1q receptor (gC1qR). The C3aR1 is expressed on several immune cells including microglia and has been implicated in various immunomodulatory processes including CNS inflammation.^12^ It is hypothesised that TLQP-21 functions as an injury signal and leads to microglial activation.^28,45^ Increased expression of TLQP-21 after peripheral nerve injury or tissue damage may contribute to neuroimmune modulation of spinal neuroplasticity. The gC1qR is expressed on both microglia and macrophages.^21^ The subsequent release of bioactive molecules may, in part, be responsible for the mechanisms of nerve-injury–induced hypersensitivity.^21,28^ The relative contribution of these 2 receptors to the physiological effects of TLQP-21 is not yet known. However, greater understanding of the role of TLQP-21, microglia, and macrophages in pain modulation will provide novel insights into pain mechanisms and has the potential to lead to the identification of novel therapeutic targets.

## 2. Objectives

The aim of this review was to identify, select, and critically appraise all relevant research, and collect and analyse data from studies investigating the role of VGF-derived peptides in pain pathways. It aims to provide a less biased summary of research findings than a conventional narrative review and allow assessment of both the range of available evidence (external validity) and the likelihood that drawn conclusions are at risk of being confounded by bias (internal validity). It will also provide direction for future research.

## 3. Methods

### 3.1. Literature search

Using the search tool within the CAMARADES-NC3Rs Preclinical Systematic Review and Meta-Analysis Facility (SyRF) and the search term “VGF,” publications were identified for screening. The platform maintains a live search and adds relevant publications as they are published. This was used to identify all relevant publications for inclusion within the review. The initial search was conducted on April 30, 2018. Subsequent updates were conducted on August 29, 2018, and July 10, 2019.

### 3.2. Screening

Studies were screened based on title and abstract by one review author (N.S.). Studies that described the role of VGF in pain were selected. Full texts of the studies were obtained on the basis that they may meet the prespecified inclusion criteria, and these comprised the systematic review data set.

### 3.3. Data extraction

#### 3.3.1. Methodological quality/risk of bias assessment

For each study detailing in vivo experimentation, quality was assessed (N.S.) against a checklist of factors relevant to animal models.^89,101^ This comprised the following criteria:

(1) Random allocation to group
(2) Allocation concealment
(3) Blinded assessment of outcome
(4) Sample size calculation
(5) Reporting of animal exclusions

In addition, journals commonly require authors to provide a statement of compliance with animal welfare regulations and declare possible conflict of interests; therefore, the reporting of these parameters was extracted too.

## 4. Results

**Figure 2.**
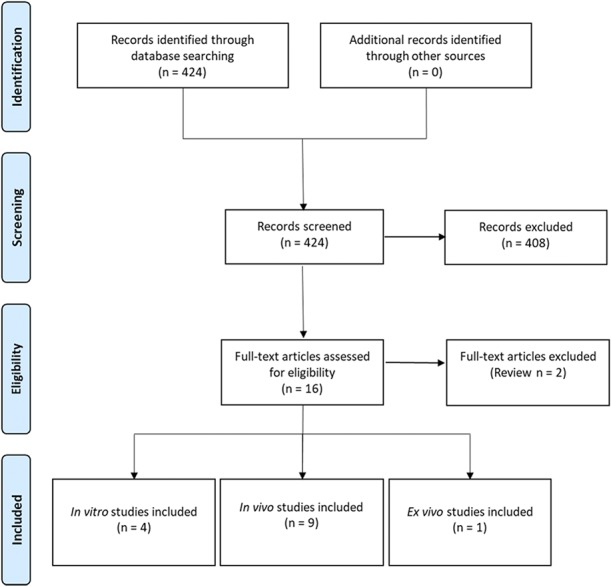
Study flow diagram. Screening and identification of publications investigating role of VGF in pain pathways. Appendix A references included studies (available at http://links.lww.com/PR9/A55).

Four in vitro studies, 9 in vivo studies, and one ex vivo study were selected for inclusion within this review (Fig. 2; Appendix A, available at http://links.lww.com/PR9/A55). Excluded records did not discuss the role of VGF in a pain context or were not primary studies. The review focuses predominantly on the data presented within the in vivo studies. Summary information and study characteristics for the included studies are reported in Tables 1–4. Spared nerve injury (SNI) is the most frequently used model to determine expression levels of both VGF mRNA and protein after nerve injury (6 studies). Six of the studies report the pronociceptive effects of administered exogenous VGF-derived peptides. TLQP-21 is the most commonly studied peptide; 5 studies assessed the effects of exogenous TLQP-21 on pain-associated behaviours. The most commonly reported outcome measure is tail-flick latency from the warm-water tail-immersion assay (3 studies).

**Table 1:**
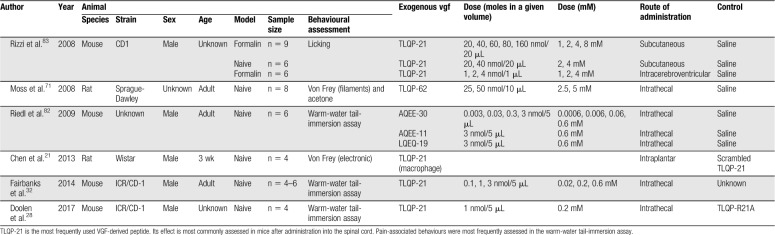
Study Characteristics for in vivo experiments assessing the effects of exogenous VGF-derived peptides.

### 4.1. Study characteristics

Six studies investigated the effect of exogenous VGF-derived peptides on pain-related behavioural outcomes (Table 1). Between the 6 studies, 4 different nerve injury models were used to assess changes in VGF expression; one study assessed changes in a model of complete Freund's adjuvant (CFA)-induced inflammation (Table 2). Four studies used antagonism to verify the role of VGF-derived peptides in potentiating pain-associated behaviours or to derive the signalling pathways (Table 3). The most frequently used behavioural assessment after administration of exogenous VGF-derived peptides was the warm-water tail-immersion assay (3 studies). Table 4 details the characteristics of the in vitro investigations in which VGF is implicated in pain signalling. Finally, multiple authors make comparisons between the different studies, and the results are summarised in Table 5.

**Table 2:**
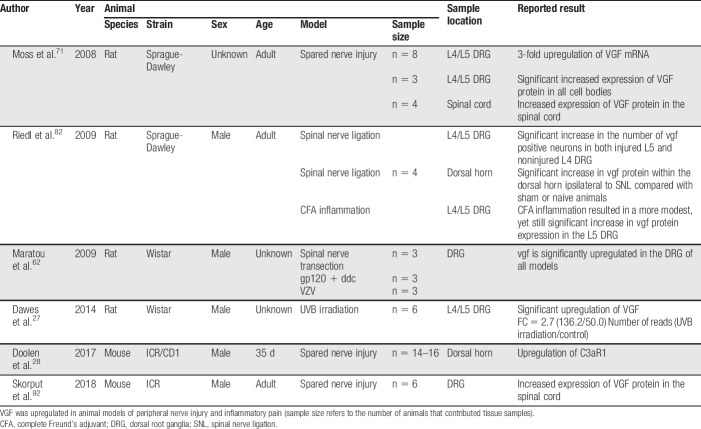
Study characteristics measuring expression of VGF in animal nerve injury models.

**Table 3:**
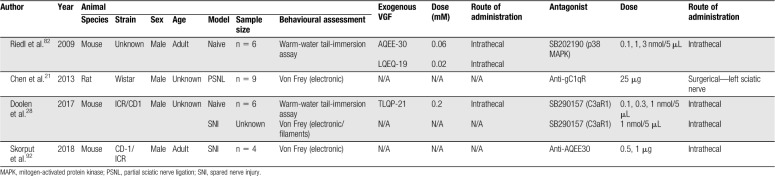
Study characteristics of in vivo experiments investigating antagonism of the effect of VGF-derived neuropeptides.

**Table 4:**
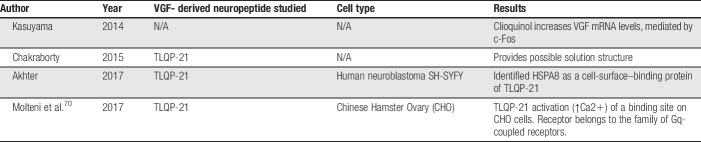
Characteristics of included in vitro studies.

**Table 5:**
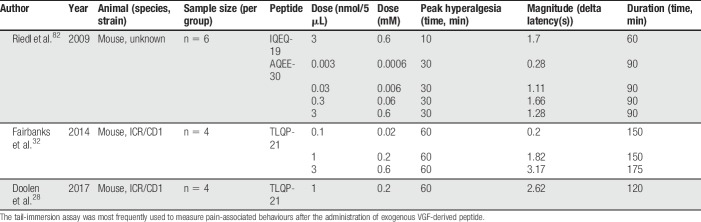
Comparison of data from the tail-immersion assay.

### 4.2. Internal validity/risk of bias

Internal validity of the 9 included studies that included animal experimentation was assessed against the reporting of 5 criteria considered relevant to animal models: blinded assessment of outcome, randomisation, allocation concealment, sample size calculation, and the reporting of animal exclusions. The mean score for the selected publications was 1 out of 5. The overall reporting of quality measures for the included publications is presented in Table 6.

**Table 6:**
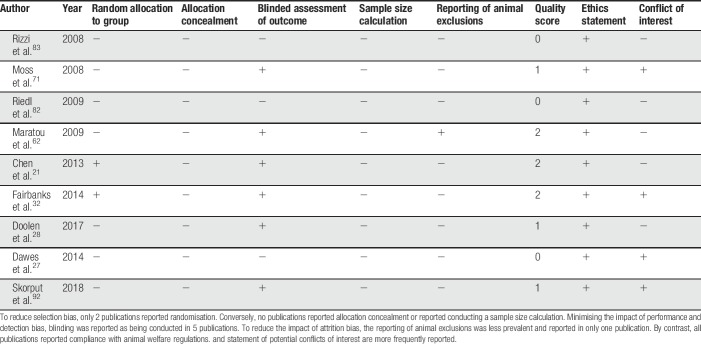
Methodological quality of studies evaluated.

## 5. Discussion/literature review

### 5.1. Localisation and upregulation of VGF

Large-scale expression analyses have been used to study the changes that occur under chronic pain conditions, leading to the identification of genes that function in pain signalling, some of which are yet to be fully characterised.^24,100,105^ The prospective significance of VGF was first derived from proteomic differential expression analysis of an in vitro model of injured sensory neurons. VGF colocalises with substance P (SP), calcitonin gene-related peptide, TrkA, and P2X3.^82,83^ Subsequent experiments provide the evidence for upregulation of VGF in the DRG and dorsal horn after nerve injury and inflammation.^27,62,71,82,92^

Moss et al.^71^ observed prolonged upregulation of VGF mRNA and protein in the DRG neurons, central terminals, and dorsal horn neurons in the SNI animal model. Injury to the sciatic nerve was associated with a 3-fold upregulation of VGF mRNA, which was maintained for 3 weeks. In addition, both the amount of VGF mRNA per cell and the number of cells expressing VGF mRNA were significantly greater in the ipsilateral compared with the contralateral DRG. At 21 days after injury, a 20-fold increase of VGF positive cells was observed in the ipsilateral compared with the contralateral DRG.

Similarly, Riedl et al.^82^ demonstrate a rapid upregulation of VGF after nerve injury and inflammation. In axotomised neurons, following 24 hours in culture, a 45-fold increase in the expression of VGF protein was detected. These changes in protein expression were paralleled in the spinal nerve ligation model corroborating earlier findings. In the L5 DRG, 24 hours after injury, the number of VGF-expressing neurons significantly increased: 80% in spinal nerve ligation animals compared with 37% in sham operated and 20% in naive animals. Interestingly, immunohistochemical analysis showed that VGF was upregulated in injured as well as uninjured neurons. The increased number of VGF neurons in DRG of sham animals suggested that VGF may also be induced in inflammatory conditions in concordance with the associated tissue damage and inflammation after surgery. This is supported by evidence, which shows that VGF is upregulated in the CFA model of inflammatory pain. Complete Freund's adjuvant inflammation resulted in a significant increase in the number of VGF-expressing neurons in the L5 DRG (28.2 ± 0.3% in CFA L5 DRG compared with 19.0 ± 2.8% in naive L5 DRG; *P* < 0.05, *t* test).

More recently, Skorput et al.^92^ provide evidence for the presence of TLQP-62 in the spinal cord and its upregulation after peripheral nerve damage. Western blot analysis of lumber spinal cord lysates from naive mice provide evidence for the bioavailability of C-terminal VGF peptides in the dorsal horn including that of TLQP-62. In concordance with other studies,^71,82^ the authors also observed a nerve-injury–induced increase in VGF levels in the DRG and spinal cord. Population analysis of anti-AQEE immunoreactivity in the DRG found increased immunoreactivity among DRG neurons of SNI mice relative to sham controls. In addition, the proportion of anit-AQEE30 labelled DRG neurons was higher in SNI mice compared with sham mice (SNI, 41.6% ± 2.2% vs sham 27.1% ± 4.0%, n = 6 per group, *P* < 0.05, unpaired Student *t* test). In addition, mice pretreated with intrathecal anti-AQEE (1 µg) immediately before peripheral nerve damage (SNI) demonstrated significantly higher paw withdrawal thresholds compared with controls in the electronic von Frey test, suggesting that VGF-derived peptides play a role in the initiation of nerve-injury–induced hypersensitivity. Similarly, attenuation of mechanical hypersensitivity was observed in a separate cohort of mice that received a single intrathecal injection of anti-AQEE30 at the time of injury.^92^

Whole-genome rat array studies were conducted by Maratou et al.^62^ to measure gene expression to identify genes that are directly relevant to neuropathic mechanical hypersensitivity. The authors compared the primary sensory neuronal gene expression profiles of 3 models of NP (peripheral nerve trauma [SNT], HIV infection, and antiretroviral-induced neuropathy and varicella zoster infection). Although these have distinct aetiologies, they share a common outcome of mechanical hypersensitivity. Comparison of the 3 models identified 39 genes to be differentially regulated in the same direction, suggesting that these genes may be responsible for the common mechanical hypersensitivity phenotype. When mechanical hypersensitivity was well established, the *vgf* gene was found to be upregulated as were NpY and Pap/Reg2 and therefore are potentially important for this phase of hypersensitivity. Similarly, Dawes et al.^27^ demonstrate that *vgf* with REG38 and CCL2 are among the most upregulated genes in the DRG in the ultraviolet B (UVB) inflammatory pain model. Riedl et al.^82^ and Moss et al.^71^ do not report measuring pain-associated behaviour before taking samples to measure VGF expression; so, a correlation cannot be drawn. However, despite the differences between the studies, the evidence is compelling: VGF expression is induced and upregulated in sensory neurons after nerve injury and inflammation. Further investigation is required to identify candidate genes that are responsible specifically for the initiation, development, and/or maintenance of NP.

Conversely, Lind et al.^57^ investigated the effects of electrical neuromodulation by spinal cord stimulation (SCS) on the cerebrospinal fluid (CSF) proteome of 14 patients responsive to SCS treatment and suggest that VGF upregulation may have an analgesic role. Most of the patients were diagnosed with radiculopathy but were a highly heterogeneous population in terms of the location of their nerve lesion(s) and where they perceive their pain. Two different proteomic mass spectrometry protocols were used to analyse the CSF samples: one taken after 48 hours of the stimulator being turned off (SCSOFF) and the second after normal use for 3 weeks (SCSON). Each patient acted as their own control. The patients in the study reported pain relief after SCS treatment, and most patients reported an increase in pain intensity scores when the device was turned off for 48 hours; neither the patients nor the investigators were blinded. In addition, there were possible confounding factors; first, the presence of paraesthesia and second, many of the patients had had lower back surgery, which made sampling challenging. Therefore, sampling may have triggered inflammation affecting the proteome. The reported results indicate that 86 CSF proteins were significantly altered in this cohort, of which VGF was in the top 12 and was increased by 38% in the SCSON condition. They report that the upregulated VGF-derived peptides were between the amino acid positions 195 and 309 corresponding to the NERP-1 chain (amino acid 281–306).^98^ The authors have not stated how they validated their findings but speculate that VGF may have active fragments that moderate or counter the biological response of their precursors, similar to that of dynorphin, angiotensin, SP, and nociceptin,^44^ and that during SCSON, there is an increase of an analgesic VGF fragment, which contributes to the long-term effects of SCS. Although NERP-1 does not overlap with the TLQP-62 peptide, from which the pronociceptive effects have been observed, the role of NERP-1 in pain pathways is unknown and changes in the expression of NERP-1 may not be related to pain.

VGF is rapidly and robustly upregulated by NGF and BDNF, which drive *vgf* gene transcription increasing VGF mRNA levels up to 50-fold in PC12 cells.^55,86^ Nerve growth factor and BDNF regulate the development and maintenance of specific functions in different populations of peripheral and central neurons. Under normal physiological conditions, NGF is synthesised by peripheral target tissues, whereas BDNF synthesis is highest within the CNS. After peripheral nerve injury, the expression of both NGF and BDNF increases rapidly.^40,68^ Peripheral nerve damage triggers NGF release by macrophages, mast cells, and Schwann cells (reviewed by Pezet and McMahon^78^), and BDNF is upregulated in tropomyosin receptor kinase A (TrkA) expressing DRG cells. TrkA is a high-affinity NGF receptor that mediates the effects of NGF. In addition, NGF/TrkA signalling has been implicated in pain pathways, particularly pain associated with inflammation.^5,61^ Anti-NGF treatment leads to reduction of hypersensitivity in peripheral nerve injury models^78^ and many of the injury-induced changes in the dorsal horn neuronal excitability are mediated by release of BDNF from microglia.^25^ In addition, BDNF is implicated in pain signalling through its interaction with the TrkB receptor.^81^ In the hippocampus, it has been demonstrated that TLQP-62 induces neuroplasticity through a BDNF-TrkB-dependent mechanism.^1,13,56,97^ TLQP-62 treatment potentiated glutamatergic responses in both the rat and mouse superficial dorsal horn.^92^ However, TLQP-62 treatment in conjunction with the Trk inhibitor K252a prevented the TLQP-62-induced increase of glutamatergic responses. These results demonstrate that the TLQP-62-induced increase in glutamatergic signalling in the dorsal horn is kinase dependent.^92^ Therefore, the upregulation of VGF in sensory neurons after nerve injury is likely to result from a spike in activity and the upregulation of these neurotrophins in the DRG and dorsal horn.^24^ The rapid and robust upregulation sets apart VGF from other peptides known to be increased after nerve injury^41,91,111^ and therefore, release of VGF peptides from sensory neurons may provide an early signal for peripheral nerve injury and abnormal pain signalling. Similarly, the sustained increase of VGF suggests that it may have a role in both the initiation and maintenance of NP.

### 5.2. Pronociceptive effects of exogenous VGF-derived peptides

Six studies reported the pronociceptive effects of exogenous VGF-derived peptides (characteristics summarised in Table 1). Most assessed central effects after intrathecal administration to naive animals (4 studies). However, 2 studies did assess the peripheral effects of specific VGF-derived peptides with either subcutaneous or intraplantar administration. The most commonly assessed VGF-derived peptide is TLQP-21 (4 studies); however, TLQP-62, AQEE-30, LQEQ-19, and AQEE-11 have also been assessed. All experiments report an increase in pain-associated behaviours that are described as increased mechanical and thermal hypersensitivity.

Rizzi et al.^83^ were the first to provide evidence for the involvement of a VGF-derived peptide in pain modulation in vivo and suggested that TLQP-21 demonstrates a different action at peripheral and central levels of the nociceptive pathway. First, peripheral injection of TLQP-21 to naive mice was associated with increased licking responses, suggesting an alteration in pain sensitivity. Second, TLQP-21 was observed to have a functional role in affecting formalin-induced pain-associated behaviour in mice. The formalin test is an acute inflammatory pain model: formalin injection to the paw induces a biphasic response. The first phase is the activation of nociceptors and corresponds to the release of peptides from nerve terminals. The second phase is posited to reflect ongoing peripheral activity and central sensitisation.^80^ In the second phase of the formalin model, the authors report that peripheral administration of the highest doses of 4- and 8-mM TLQP-21 results in worsening pain-associated behaviours (increased licking responses), thereby suggesting that TLQP-21 may have a functional role in the inflammatory process induced by formalin. In support of TLQ-21 having a functional role in the inflammatory process, intraplantar injection of macrophages stimulated with TLQP-21, likely mediated through the gC1q receptor, evokes mechanical hypersensitivity in rats.^21^

Conversely, intracerebroventricular (i.c.v) administration of 2 nM/1 µL/mouse TLQP-21 resulted in reduced licking response in the second phase, which the authors describe to be an analgesic effect^83^; however, the lower dose of 1 mM and the highest dose of 4 mM did not have an effect. The authors postulate that TLQP-21 modulation of inflammatory pain depends on the route of administration and that the analgesic effect of central administration could be a consequence of a modulatory role exerted on the descending inhibitory pathway of which the glutamatergic, adrenergic, and serotonergic systems could be the target of its action.^69^ However, this analgesic phenomenon does not seem to have been investigated further to provide confirmatory or concomitant evidence.

Acute intrathecal administration of TLQP-62 (2.5 and 5 mM) to naive rats was associated with both mechanical (von Frey filaments) and cold (cutaneous acetone) behavioural hypersensitivity.^71^ Similarly, intrathecal administration of both AQEE-30 and LQEQ-19^82^ and TLQP-21^28^ to naive mice, dose-dependently induced thermal hypersensitivity assessed in the warm-water tail-immersion assay.

Authors have made comparisons about the magnitude and duration of effect of the VGF-derived peptides with TLQP-21 being described as having the greatest magnitude and duration of effect in the warm-water tail-immersion assay (Table 5). However, the peptides were not assessed in the same experiment and therefore a prospective experiment is required to directly compare their actions and dose response to allow for conclusions to be drawn about magnitude and duration of effect.

Riedl et al.^82^ also evaluated several pharmacological agents known to interfere with pathways involved in nociceptive processing to determine whether they can inhibit the AQEE-30- and LQEQ-19-induced thermal hypersensitivity. These included inhibitors of nitric oxide synthase (7-NI, 10 nM; l-NAME, 100 nmol), NMDA receptor (MK801, 10 nmol), protein kinase C (GF109203X, 1 and 10 nmol), protein kinase A (KT5720, 1.7 nmol), and mitogen-activated protein kinase (MAPK) (U0126, 2.5 nmol; SB202190 0.1, 1 and 2.5 nmol; SB600125, 2.5 nmol). It is reported that only the p38 inhibitor SB202190, administered as a pretreatment, dose-dependently reversed AQEE-30 (0.3 nmol) and LQEQ-19 (1 nmol) evoked thermal hypersensitivity (data of other inhibitors not shown), suggesting that the effects of VGF-derived peptides are mediated by p38. In corroboration of these findings, Fairbanks et al.^32^ demonstrated TLQP-21 evoked p-38 MAPK-dependent thermal hypersensitivity. The effects of intrathecal TLQP-21 were attenuated by treatment with either the p38 inhibitor (SB202190), COX inhibitor (indomethacin), or the lipooxygenase inhibitor (AA-861) assessed in the warm-water tail-immersion assay.

The in vivo evidence suggests that VGF-derived peptides may be involved in both nociception and inflammatory pain. VGF may have a direct or indirect action that leads to the release of other neuropeptides and neurotransmitters. It has been shown in cultured DRG cells that VGF-derived peptides are colocalised with SP.^33^ It is well established that SP has a role in nociception^6,76,106^ and in the pathophysiology of inflammatory disease.^74^ VGF-derived peptides therefore may be part of the mixture of molecules produced by the DRG neurons, which are secreted in response to nociceptive stimuli together with the release of SP,^60^ hence their proposed role in the inflammatory process. TLQP-21 stimulates COX activity^32^ leading to the synthesis of prostaglandins, important mediators of inflammation and pain. Prostaglandins increase levels of cyclic AMP and enhance nociceptor sensitisation by reducing the activation threshold of sodium channels^31^ as well as sensitising primary afferent neurons to bradykinin and other mediators.^73^

### 5.3. Identification of receptors, their expression, and their role in pronociceptive signalling

Despite the large volume of data on physiological effects of VGF-derived peptides, very little is known about the receptors with which VGF-derived peptides interact, their downstream signalling pathways, and mechanisms of action. It has been hypothesised that VGF-derived peptides may lead to the sensitisation of sensory neurons by direct (autocrine) or indirect (paracrine) action. The complement component 3a receptor (C3aR)^45,70^ and globular head of the complement component C1q receptor (gC1qR)^21^ have been identified as receptors for TLQP-21; the receptors for the other VGF-derived peptides have not been identified. It is unanimously reported that TLQP-21 treatment leads to increased intracellular Ca^2+^ release. Ca^2+^ is a highly versatile second messenger involved in a variety of intracellular signalling pathways, including gene regulation, cell proliferation, and death.

#### 5.3.1. The globular head of the complement component C1q receptor (gC1qR)

Chen et al.^21^ report that gC1qR is expressed on both macrophages and microglia and the peripheral pronociceptive actions of TLQP-21 involve the activation of gC1qR on macrophages. *In vitro*, they demonstrated that TLQP-21 treatment (not TLQP-62 or LQEQ-19) is associated with an increase in intracellular Ca^2+^ levels in cultured rat bone-marrow–derived primary macrophages and brain-derived primary microglia. However, TLQP-21 did not induce changes in intracellular Ca^2+^ levels in cultured DRG neurons, suggesting that TLQP-21 does not directly hypersensitise sensory neurons (authors' did not publish the data), supporting the hypothesis that TLQP-21 effect is mediated by receptors expressed on macrophages and microglia.^21^ They also demonstrate that this increase in intracellular Ca^2+^ levels in macrophages is gC1qR dependent. Silencing of gC1qR with siRNA in macrophages significantly reduced the gC1qR protein expression, and fewer macrophages responded to TLQP-21 treatment. Similar results were observed after preincubation of macrophages with neutralising gC1qR monoclonal antibodies resulting in significant reduction in response to TLPQ-21.^21^

Chen et al.^21^ also examined the role of gC1qR in pain pathways in vivo: first, intraplantar injection of TLQP-21-stimulated macrophages resulted in mechanical hypersensitivity in rats. Second, the gC1qR antibody (Mab1) was applied to the site of nerve ligation in the partial sciatic nerve ligation model rats. Application of the gC1qR antibody delayed the onset of mechanical hypersensitivity associated with partial sciatic nerve ligation, whereas control IgG-treated rats demonstrated a reduction of punctate mechanical threshold. Therefore, macrophages stimulated by TLQP-21 through gC1qR may be responsible for the hypersensitivity of sensory neurons, characteristic of the mechanical hypersensitivity observed.

#### 5.3.2. The complement component 3a receptor (C3aR1)

Hannedouche et al.^45^ first described the identification of C3aR1 as a target for TLQP-21 in rodents. Doolen et al.^28^ provide functional and anatomical evidence that the spinal pronociceptive actions of TLQP-21 involve the activation of C3aR1 of microglia in the dorsal horn. They demonstrate extensive C3aR1 colocalisation with Iba-ir microglia in the dorsal horn (Iba1; a marker for macrophages and microglia). Their colocalisation analysis of C3aR1, GFAP, and NeuN labelling did not yield evidence for C3aR1 expression in astrocytes or neurons; therefore, TLQP-21 directly targets C3aR1 to activate spinal microglia rather than neurons. They also demonstrate that in Iba1-eGFP mice, TLQP-21 elicits Ca^2+^ signalling in microglia with over 95% of eGFP-positive profiles responding to TLQP-21, suggesting that the Ca^2+^ release in response to TLQP-21 is a result of microglial activation. In addition, in C3aR1 knockout mice and wild-type cultures exposed to R21A (an inactive peptide at C3aR1; a single amino-acid substitution in the C-terminus of TLQP-21),^20^ treatment with TLQP-21 did not lead to an increase in intracellular Ca^2+^ signalling, further reinforcing the evidence that TLQP-21 signalling is C3aR1 mediated.^28^

Doolen et al.^28^ also confirm in vivo the molecular and structural findings that TLQP-21 directly activates C3aR1.^19,45^ Peripheral nerve injury (SNI) increased both microglial C3aR1 expression and TLQP-21-evoked C3aR1-mediated Ca^2+^ signalling in the dorsal horn of the spinal cord. Spared nerve injury also increased both the number of microglia with increased intracellular Ca^2+^ signalling and the peak amplitude, indicating that nerve injury results in increased TLQP-21 signalling. Pharmacological inhibition of the C3aR1 with the antagonist SB290157 attenuated the hypersensitivity associated with SNI. In microglial cell culture, the increased Ca^2+^ signalling after treatment with TLQP-21 was also attenuated. Together, these results provide the evidence that microglial C3aR1 signalling is required for nerve-injury–induced hypersensitivity.^28^

**Figure 3.**
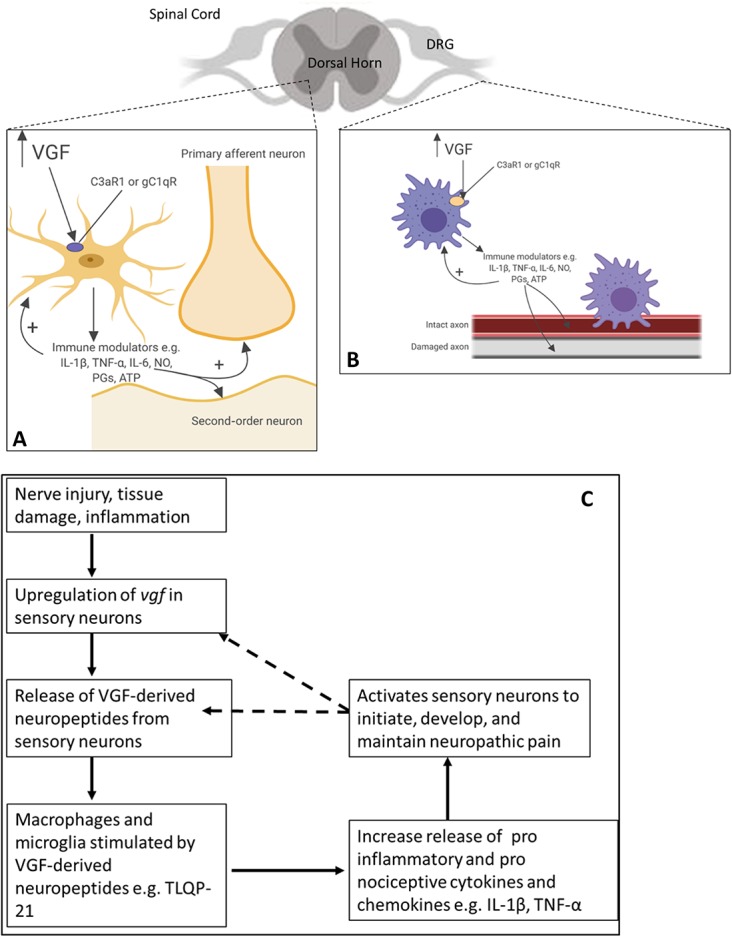
The role of VGF-derived neuropeptides in (A) central and (B) peripheral sensitisation and (C) neuropathic pain. (A) Microglia in the spinal dorsal horn are activated by VGF-derived neuropeptides after nerve injury through their interaction with either or potentially both the C3a1 or gC1q receptors. In turn, these activated microglia release several proinflammatory cytokines, chemokines, and other agents, which interact with neurons associated with pain transmission and increase the excitability of the neurons contributing to NP. Proinflammatory mediators released, eg, TNFα, IL-1β, IL-6, nitric oxide (NO), ATP, and prostaglandins (PGs), initiate a self-propagating mechanism of enhanced cytokine expression by microglial cells. This leads to an increase in intracellular calcium, and activation of the p38 and MAPK pathway. (B) Damaged and spared fibres are pictured together. After peripheral nerve injury, nonneuronal cells including macrophages accumulate around the damaged cells. The upregulation and release of VGF-derived neuropeptides activate the macrophages through their interaction with either or potentially both the C3a1 or gC1qR receptors. The macrophages secrete factors (such as TNFα, IL-1β, IL-6, chemokine [C–C motif] ligand 2 [CCL2], PGs, and nerve growth factor [NGF]), which elicit peripheral sensitisation and cause chronic neuroinflammation, which maintains sensory abnormalities. (A and B) were created with Biorender.com. (C) The graph abstract highlights the potential role of *vgf* and VGF-derived peptides in the initiation, development, and maintenance of neuropathic pain. MAPK, mitogen-activated protein kinase; NP, neuropathic pain.

Despite the identification of 2 receptors, the signalling mechanisms of VGF-derived peptides are completely uncharacterised. Findings to date describe a receptor within the CNS (C3aR1) that is activated by both a peptide (TLQP-21) and an immune mediator (C3a), which is mirrored in the periphery with gC1qR and C1q. Interestingly, gC1qR and C3aR1 are receptors for the complement proteins C1q and C3a, respectively. They form part of the complement system that consists of enzymes, regulatory proteins, and receptors that regulate both innate and adaptive immune responses. It is possible that TLQP-21 interacts with both receptors. Because the TLQP-21 precursor VGF is upregulated in sensory neurons within 24 hours of nerve injury,^82^ it is probable that TLQP-21 released from injured sensory neurons functions as a warning or protective signal that contributes to spinal neuroinflammation through activation of C3aR1 and peripheral neuroinflammation through gC1qR. It has not yet been investigated whether the gC1qR expressed on microglia in the CNS and whether C3aR1 expressed on macrophages in the periphery also play a role in TLQP-21/VGF-derived peptide signalling; however, the identification of these 2 receptors suggests a novel interplay of neuronal and immune signalling mediators in both peripheral and CNS diseases (Fig. 3).

#### 5.3.3. Pronociceptive signalling

Our understanding of pain signalling traditionally has focused on the neuronal system; however, this is neurobiologically incomplete, and immune cells have an important role as pain modulators.^63,88,96^ Macrophages play key roles in the complement effector functions, and the involvement of macrophages in NP pathogenesis has been well reviewed.^11,16^ Maratou et al.^62^ identified Pap/Reg2, a macrophage chemoattractant, as another molecule commonly upregulated in the 3 models of NP. Resident macrophages in the DRG proliferate after nerve injury,^72^ and circulating monocytes are recruited to the site of injury. Depletion of macrophages reduces mechanical hypersensitivity after nerve injury and, in an animal model of diabetic neuropathy, delays the onset and progression of pain-associated behaviours.^58,67^ Macrophages and monocytes express a variety of receptors, including both gC1qR and C3aR1, and activation of these receptors leads to the modulation of cytokine production and inflammatory responses.

There is evidence to suggest that spinal microglia participate in the development of inflammatory and nerve-injury–induced hypersensitivity, perhaps in part through the activation of p38 MAPK. Mitogen-activated protein kinases are a family of evolutionally conserved molecules that play a critical role in cell signalling. Together, they transduce a broad range of extracellular stimuli into diverse intracellular responses by both transcriptional and nontranscriptional regulation.^50,75,109^ They are activated by upstream kinases through phosphorylation.^3,51,85^ Riedl et al.^82^ demonstrated that the VGF-derived peptides AQEE-30 and LQEQ-19 activate p38 MAPK in mouse microglia. Immunohistochemical analysis of p38 MAPK activation in spinal cords obtained at the time of peak hypersensitivity from mice injected intrathecally with LQEQ-19 measured a near 3-fold increase in phosphorylated p38 MAPK. The role of p38 MAPK has also been assessed in vivo. TLQP-21-induced thermal hypersensitivity was inhibited dose-dependently by the potent and selective p38 MAPK inhibitor SB202190.^32^ After nerve injury or inflammation, the combination of VGF upregulation and increased excitability of sensory neurons is likely to potentiate the release of VGF-derived peptides within the dorsal horn, in which they would be able to activate microglial p38 MAPK.

In addition, C3aR1 activation of monocytes induces release of ATP and cytokines.^4^ Astrocytes in the spinal cord also have the capacity to release ATP,^107^ and microglial purinergic receptors are heavily implicated in NP mechanisms.^10^ It is therefore plausible that stimulation of macrophages and/or microglia with TLQP-21 may lead to production and secretion of proinflammatory cytokines through a gC1qR- or C3aR1-MAPK-dependent pathway.^23,43,82^ In turn, these cytokines may be responsible for the hypersensitisation of the sensory neurons.

Both Chen et al.^21^ and Doolen et al.^28^ demonstrated attenuation of mechanical hypersensitivity in nerve injury models after inhibition of gC1qR or C3aR1, respectively. It is not clear what the specific roles of the receptors are and whether both are responsible and/or have a role to play in both the peripheral and central systems. However, together, these findings suggest that macrophages and/or microglia stimulated by TLQP-21 through either gC1qR and/or C3aR1 may have a role to play in the initiation, development, and maintenance of NP and that disrupting TLQP-21 receptor interaction or its downstream signalling pathway may be a viable strategy for pain management.

### 5.4. Technical limitations

#### 5.4.1. Animal models of neuropathic pain (predictive value) and behavioural outcomes

Animal experimentation has contributed extensively to our understanding of mechanisms of disease and development of novel therapeutics; however, their predictive value of treatment effectiveness in humans remains controversial. Despite promising preclinical evidence, the lack of success in the clinic suggests that there are limitations in their translatability.^77^ Pain is a subjective, multifaceted symptom that can only be measured by self-reporting,^102^ and the presence or absence of pain in animal models cannot be directly measured and can only be inferred from the observation of surrogate behaviours.^30^ In the assessment of VGF-derived peptide expression, SNI is the most commonly used model (5 studies). Neuropathic pain is highly heterogeneous, both within and across underlying conditions, and the predominance of traumatic nerve injury models does not match the clinical situation.^37^ A more rational approach is to select models that more closely reflect the pathophysiological condition of humans. Hence, disease-specific models have been developed including models of varicella-zoster virus infection^39,42,46^ and HIV-associated peripheral neuropathy.^103,104^ To add, reflex withdrawal assessments do not necessarily measure global pain and therefore can be misleading, if not just a measure of nociception. Predictive validity may also be improved by measuring changes in behaviours that are ethologically relevant to rodents and may be affected by pain, eg, rearing,^64^ feeding,^95^ and burrowing.^2^

#### 5.4.2. Internal validity

Characteristic of the field, the lack of reporting of methods undertaken to reduce the risk of bias suggests that the in vivo data reported in the studies within this review may be susceptible to bias. Experimental bias is often unintentional and can be because of low internal validity leading the scientist to incorrectly attribute an observed effect to a treatment or intervention.^47^ Internal validity ensures that changes observed in outcomes are due to an induced change in the independent variable rather than confounding factors. The internal validity may be compromised by a range of biases: selection, performance, detection, and attrition bias. There are several mitigations that will reduce the risk of these biases; randomisation, allocation concealment, sample size calculation, blinded assessment of outcome, and a predetermined animal eligibility criterion. Several systematic reviews and meta-analyses have provided empirical evidence highlighting that inadequate experimental approaches are associated with bias in several preclinical fields.^26,48,84^ Of concern is the fact that low prevalence of reporting of measures to reduce the risk of bias tend to give higher estimates of treatment effects.^26,48^ The introduction of the ARRIVE reporting guidelines in 2010^52,53,65^ and the development of National Centre for the Replacement, Refinement and Reduction of Animals in Research Experimental Design Assistant gives preclinical researchers clear guidance on how to conduct animal experiments with appropriate rigour.^29^

In addition, as is often the case, the publications included in the review may be susceptible to publication bias. TLQP-21 is the most frequently researched VGF-derived peptide, particularly in recent years. It was not possible to estimate publication bias due to the low number of studies. However, publication bias deprives researchers of accurate data that are needed to generate new hypotheses and prevent a waste of resources in the case that a direction of research is chosen that has already been fully exploited but not published due to neutral or negative data. Thus, there is a critical and ethical need for transparency of reporting all experimental details to complete the story.^90^

## 6. Conclusion and recommendations for future research

A persistent challenge in the management of NP is to target the specific mechanisms leading to a change from normal to abnormal sensory perception while ensuring that the defensive pain perception remains intact. Targeting VGF-derived peptides may offer this opportunity. Peripheral tissue injury is associated with changes in protein expression in sensory neurons that may contribute to abnormal nociceptive processing. The publications within this review have focused on exploring the role of VGF-derived peptides in vivo but the expression of VGF in NP patients has not been characterised. The presumed abundant and selective expression of the VGF-derived peptides in blood and CSF suggest a possibility that they could be used as biomarkers of NP. The understanding of the molecular mechanisms and signalling events by which VGF-derived active peptides exert their many physiological actions is in its infancy. Future work should aim to have a better understanding of the downstream consequences of cell treatment with TLQP-21, which should uncover proteomic changes in TLQP-21-treated cells and intracellular mechanisms of TLQP-21 actions, and subsequent understanding may offer therapeutic strategies for NP. The identification of the 2 complement receptors with natural ligands, of which little is understood, suggests the possibility of a dual-ligand mechanism of action. There are both central and peripheral mechanisms to VGF-derived peptide activity; yet, it is not clear whether the mechanisms are distinct and/or have different roles in the initiation, development, and maintenance of NP. Given that VGF expression levels are very low under normal physiological conditions suggests that it does not play a role in nociception but its rapid upregulation in sensory neurons after nerve injury and inflammation along with the activation of microglia and macrophages may in part be responsible for the onset, development, and maintenance of neuropathic and inflammatory pain.

## Disclosures

K. Okuse: an inventor on patents: Okuse K. et al. Methods of treating pain by inhibition of vgf activity EP13702262.0/WO2013 110945, and Okuse K, Ayub M, Swanwick R, et al., 2013, New receptor identified for neuropathic pain; A.S.C. Rice: reports consultancy and advisory board work for Imperial College Consultants in the last 24 months; this has included remunerated work for: Galapagos, Toray, Quartet, Lateral, Novartis, Pharmaleads, Cambridge University (Prof Peter McNaughton), Orion, Asahi Kasei, and Theranexus, outside the scope of the submitted work. In addition, A.S.C. Rice was the owner of share options in Spinifex Pharmaceuticals from which personal benefit accrued on the acquisition of Spinifex by Novartis in July 2015 and from which future milestone payments may occur. In addition, Dr Rice is named as an inventor on patents: Rice A.S.C., Vandevoorde S. and Lambert DM. Methods using N-(2-propenyl)hexadecanamide and related amides to relieve pain. WO 2005/079771, and Okuse K. et al. Methods of treating pain by inhibition of vgf activity EP13702262.0/WO2013 110945; the remaining author has nothing to declare.

The work is supported by the BBSRC (grant number BB/M011178/1).

## Appendix A. Supplemental digital content

Supplemental digital content associated with this article can be found online at http://links.lww.com/PR9/A55.
